# Alcoholic Hepatitis in a Japanese Hospital: Losing Contact With Some Patients After Delirium Tremens May Lead to Missed Critical Events

**DOI:** 10.1111/adb.70052

**Published:** 2025-06-02

**Authors:** Hisanori Muto, Teiji Kuzuya, Yoshihiko Tachi, Yoshiaki Katano, Naoki Ohmiya, Takashi Kobayashi, Satoshi Yamamoto, Naoto Kawabe, Hijiri Sugiyama, Seiya Hagihara, Misae Matsushita, Yutaro Kajino, Yosuke Nagano, Senju Hashimoto

**Affiliations:** ^1^ Department of Gastroenterology and Hepatology Fujita Health University Bantane Hospital Nagoya Japan; ^2^ Department of Gastroenterology and Hepatology Fujita Health University Toyoake Japan; ^3^ Department of Gastroenterology and Hepatology Fujita Health University Okazaki Medical Center Okazaki Japan; ^4^ Department of Advanced Endoscopy Fujita Health University Toyoake Japan

**Keywords:** acute‐on‐chronic liver failure, alcohol withdrawal syndrome, alcoholic hepatitis, benzodiazepine, delirium tremens

## Abstract

In Japan, the establishment of diagnostic criteria for acute‐on‐chronic liver failure (ACLF) in 2022 has increased the focus on alcoholic hepatitis. Most hospitals in Japan lack specialized treatment units or psychiatrists for managing alcohol use disorders, leaving hepatologists to handle various aspects of the disease—a challenging task. This study retrospectively investigated the outcomes of alcoholic hepatitis in a typical Japanese hospital setting, stratified by ACLF diagnosis and other features, with the aim of identifying areas for possible improvement. We conducted a retrospective analysis of 88 patients hospitalized with alcoholic hepatitis, reviewing records for the diagnosis of ACLF or related conditions, development of delirium tremens (DT), risk factors, and patient outcomes. Patients meeting the Japanese criteria for ACLF or related conditions had significantly worse survival outcomes. DT developed in 13 patients, with low platelet counts and elevated γ‐glutamyl transpeptidase levels identified as risk factors. Prophylactic oral benzodiazepines were found safe and significantly associated with preventing DT. Onset of DT during hospitalization did not measurably impact survival prognosis, but DT patients showed a tendency to break contact with our hospital and critical events may have been missed. While under hepatologist care, patients typically maintained sobriety, but relapse into alcohol‐related health problems frequently occurred after follow‐up was discontinued. In Japan, hepatologists may be missing important events with alcoholic hepatitis after follow‐up discontinuation, especially in patients with DT. Therefore, integrated and collaborative care, particularly a psychosocial approach providing behavioural support, may reduce risk of relapse and improve patient prognosis.

**Trial Registration:**

All study protocols were reviewed and approved by the ethics committee at Fujita Health University School of Medicine (approval no. HM23‐213)

## Graphical abstract

In a Japanese hospital without psychiatric support, 15% of alcoholic hepatitis patients developed delirium tremens (DT), which shortened follow‐up. Prophylactic oral benzodiazepines were safe and significantly reduced the risk of DT. Integrated care models are needed to prevent missed critical events and relapses after discharge.

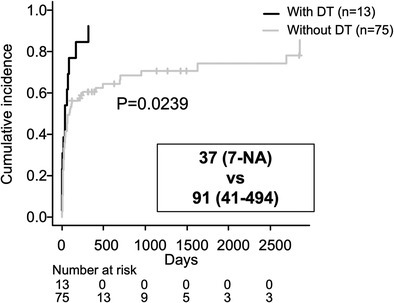

**Abbreviations**
ACLF: acute‐on‐chronic liver failure
DT: delirium tremens
ASAM: American Society of Addiction Medicine
MELD: Model for End‐stage Liver Disease
JAS: Japan Alcoholic Hepatitis Score
CI: confidence interval
Hb: haemoglobin
WBC: white blood cell
PLT: platelet
INR: international normalized ratio
AST: aspartate aminotransferase
ALT: alanine aminotransferase
GGT: γ‐glutamyl transpeptidase
T.Bil: total bilirubin
BUN: blood urea nitrogen
Cre: creatinine
Alb: albumin
PAWSS: Prediction of Alcohol Withdrawal Severity Scale
CIWA‐Ar: Clinical Institute Withdrawal Assessment scale for Alcohol, revised

## 1 Introduction

Between 2018 and 2021 in Japan, alcohol‐related liver disease surpassed viral hepatitis as the leading cause of cirrhosis [1]. In 2022, diagnostic criteria for acute‐on‐chronic liver failure (ACLF) were established in Japan [2], revealing that most ACLF cases are linked to severe alcoholic hepatitis [3]. However, the natural history and prognosis of these cases in clinical practice remain poorly understood.

A nationwide survey by the Japanese Ministry of Health, Labour and Welfare revealed that out of 7100 general hospitals providing internal medicine care, only 1800 (25.4%) also provide psychiatric care [4]. Despite this, little is known about the current state of alcoholic hepatitis treatment in general hospitals without psychiatry, especially regarding the management of DT and follow‐up.

In Japan, approximately 1.0% of the population (1.9% of men and 0.2% of women) are diagnosed with alcohol abuse according to the International Statistical Classification of Diseases and Related Health Problems, 10th Revision, with a particularly high incidence of severe alcohol‐related liver disease [5]. Withdrawal symptoms often occur during hospitalization after the sudden cessation of heavy alcohol consumption, and managing these symptoms becomes particularly challenging when they escalate into delirium tremens (DT) [6, 7]. However, the treatment of DT and its impact on the prognosis and follow‐up of alcoholic hepatitis remain poorly understood. Symptoms of autonomic excitability, such as sweating, tachycardia and fever, tremors, anxiety, irritability and insomnia, typically appear from 6 h after sobriety [8]. Severe withdrawal symptoms can progress to DT, which is characterized by impaired consciousness and visual and auditory hallucinations, and benzodiazepine treatment is often used to prevent this development [9].

The present study was performed in our hospital, which represents a typical Japanese hospital where hepatologists are responsible for all aspects of alcoholic hepatitis management, without the help of specialized staff. As this was considered to be a potential area for improvement, we focused on alcohol withdrawal symptoms, which are common in alcoholic hepatitis and ideally should be managed with the support of a psychiatrist [8]. We conducted a retrospective analysis of patients hospitalized with alcoholic hepatitis, reviewing records for diagnosis of ACLF or related conditions, development of DT, risk factors, and patient outcomes.

## 2 Methods

### 2.1 Patients

The retrospective study included 88 Japanese patients who were negative for Hepatitis B surface antigen and hepatitis C virus antibodies, regularly consumed alcohol (40 g or more per day for women and 60 g or more per day for men) [7], were admitted to Fujita Medical University Bantane Hospital between January 2013 and April 2024, and were clinically diagnosed with alcoholic hepatitis. All study protocols were approved by the ethics committee of Fujita Health University School of Medicine and were conducted in accordance with the 1975 Declaration of Helsinki (approval no. HM23‐213). Written informed consent for treatment was obtained from each patient, though informed consent was not required for participation in this study because of the retrospective design.

### 2.2 Treatment

The treatment of alcoholic hepatitis included abstinence and supportive care, and, in some cases, administration of prednisolone or methylprednisolone [6, 9]. The prevention of DT was managed with diazepam at doses of 4 to 12 mg/day or lorazepam at doses of 1.5 to 3 mg/day, at the discretion of the attending physician. This preventive treatment was typically given to patients considered at high risk for DT based on their history of heavy drinking, their history of alcohol withdrawal symptoms, the results of alcohol dependence screening tests such as CAGE [10] and AUDIT [11], and the presence or absence of autonomic nervous system symptoms such as sweating and tachycardia, in accordance with the American Society of Addiction Medicine (ASAM) guidelines [12, 13, 14]. If DT nevertheless developed, antipsychotics (haloperidol or risperidone) and continuous sedation with midazolam or propofol were administered as appropriate. Because Fujita Medical University Bantane Hospital did not have a psychiatrist on staff, all treatment decisions, including those related to DT prevention and management, were made between the patient and the attending hepatologist based on informed consent. Although drug therapies for the prevention and treatment of DT were administered with reference to the ASAM guidelines, the absence therein of clear criteria for preventive treatment and specific dosage recommendations often left actual treatment decisions to the attending physician.

### 2.3 Follow‐Up and Statical Analysis

The follow‐up period was defined as the interval from the start of inpatient treatment to the date of the last hospital visit, when follow‐up was discontinued either by the attending physician's decision or by the patient without authorization. The observation period was defined as the interval from the start of inpatient treatment to the last date on which the patient's survival was confirmed in the medical record. ACLF or related conditions were diagnosed according to the criteria established by the Intractable Hepato‐Biliary Disease Study Group in Japan [2]. The severity of alcoholic hepatitis was rated by the Japan Alcoholic Hepatitis Score (JAS) [15]. Statistical analysis was performed using Easy R (EZR) version 1.61 (Saitama Medical Center, Jichi Medical University, Omiya, Japan) [16]. Factors with *p* < 0.1 in univariate analysis were included in multivariate analysis. Factors with *p* < 0.05 were considered significant.

## 3 Results

### 3.1 Baseline Characteristics

The baseline characteristics of the patients are shown in Table 1. The median age was 54.5 years. Of the 88 patients, 68 (77.3%) were male. A total of 34 patients (38.6%) met the criteria for ACLF or related conditions diagnosis, while 26 had an unknown baseline Child–Pugh score and were therefore classified as ‘probable’ or ‘extended probable’. A JAS score of 8 or higher was found in 38 patients (43.2%). Steroids were administered to 8 patients (9.1%) as part of their treatment for severe alcoholic hepatitis, and 50 patients (56.8%) received benzodiazepines to prevent DT.

**TABLE 1:** Baseline characteristics. *n* (%), median (IQR).

Sex (male/female)	68 (77.3)/20 (22.7)
Age	54.5 (44.75–65)
Diagnostic criteria for ACLF (ACLF/Extended/Probable/Extended Probable/without ACLF)	6 (6.8)/2 (2.3)/7 (8.0)/19 (21.6)/54 (61.4)
JAS (≥ 8/≤ 7)	38 (43.2)/50 (56.8)
Gastrointestinal bleeding (yes/no)	7 (8.0)/81 (92.0)
Ascites (yes/no)	28 (31.8)/60 (68.2)
Hb (g/dL)	11.65 (10.2–13.53)
WBC (/μL)	7200 (5200–10 350)
PLT (×10^3^/L)	135 (88–192)
INR	1.23 (1.00–1.47)
AST (U/L)	163 (112.25–292.5)
ALT (U/L)	59.5 (35.75–148.5)
GGT (U/L)	564 (305.25–1056.25)
T.Bil (mg/dL)	2.45 (1.48–5.43)
BUN (mg/dL)	11 (7–16.25)
Cre (mg/dL)	0.74 (0.62–1.05)
Alb (g/dL)	3.3 (2.7–3.9)
MELD	12 (9–18)
Benzodiazepine prophylaxis (yes/no)	50 (56.8)/38 (43.2)
Administration of prednisolone or methylprednisolone (yes/no)	8 (9.1)/80 (90.9)

Abbreviations: ACLF, acute‐on‐chronic liver failure; Alb, albumin; ALT, alanine aminotransferase; AST, aspartate aminotransferase; BUN, blood urea nitrogen; Cre, creatinine; DT, delirium tremens; GGT, γ‐glutamyl transpeptidase; Hb, haemoglobin; INR, international normalized ratio; JAS, Japan Alcoholic Hepatitis Score; MELD, Model for End‐stage Liver Disease; PLT, platelet; T. Bil, total bilirubin; WBC, white blood cell.

### 3.2 Association Between the Diagnosis for ‘ACLF or Related Conditions’ and Survival Prognosis

**FIGURE 1:**
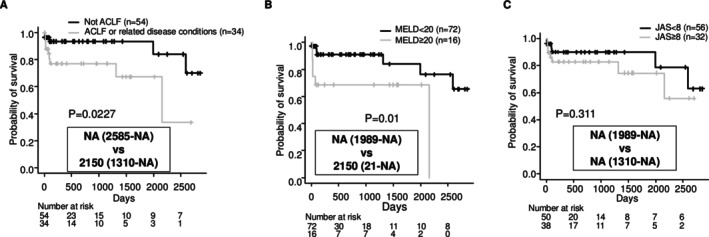
Kaplan–Meier survival curves based on whether patients meet specific diagnostic criteria. Survival curves stratified by whether patients meet the diagnostic criteria for ACLF. The MST was NA in the group meeting the ACLF criteria, with a 95% CI of 2585 days to NA. In the group not meeting the criteria, the MST was 2150 days (95% CI 1310 days to NA). The log‐rank test yielded a *p* value of 0.0227. Survival curves stratified by whether the (MELD score was ≥ 20 or < 20. The MST in the group with MELD ≥20 was NA, with a 95% CI of 1989 days to NA. For patients with MELD < 20, the MST was 2150 days (95% CI 21 days to NA). The log‐rank test yielded a *p* value of 0.01. Survival curves stratified by whether the JAS score was ≥ 8 or < 8. The MST in the group with a JAS score ≥ 8 was NA, with a 95% CI of 1989 days to NA. In the group with a JAS score < 8, the MST was NA, with a 95% CI of 1310 days to NA. The log‐rank test yielded a *p* value of 0.311. Note: NA (indicates that the median survival time was not reached within the observation period. MST, median survival time; NA, not applicable; CI, confidence interval; ACLF, acute‐on‐chronic liver failure; MELD, Model for End‐stage Liver Disease; JAS, Japan Alcoholic Hepatitis Score.

Kaplan–Meier survival curves, distinguished by negative and positive diagnoses for ACLF or related conditions, are shown in Figure 1A. Patients with ACLF or related conditions had a significantly worse survival prognosis (median survival: NA vs. 2150 days, *p* = 0.0227). Prognosis was also stratified by MELD score (Figure 1B). However, no significant stratification of prognosis was found according to whether patients had a JAS score of 8 or higher (Figure 1C). Patients with ACLF or related conditions had significantly higher peripheral white blood cell counts, and the proportion of these patients with a JAS score of 8 or higher was much higher (Table 2). All patients treated with steroids were those with ACLF or related conditions (Table 2). Six patients underwent pulse therapy with methylprednisolone at doses ranging from 500 to 1000 mg, while two patients were started on oral prednisolone at doses of 35 to 40 mg. There were 14 deaths during the observation period, with causes of death including liver failure (7 cases), hepatocellular carcinoma (2 cases), and other causes (5 cases: pneumonia, arrhythmia, intracranial haemorrhage, stomach cancer, and traffic accident).

**TABLE 2:** Baseline characteristics by ACLF diagnostic criteria. *n* (%), median (range). Fisher's exact test was performed for categorical variables and Mann–Whitney's *U* test for continuous variables.

	ACLF or related conditions (*n* = 34)	Without ACLF (*n* = 54)	*p* value
Sex (male/female)	25 (73.5)/9 (26.5)	43 (79.6)/11 (20.4)	0.724
Age	51 (44.25–63.75)	55.5 (45–66.5)	0.874
JAS (≥ 8/≤ 7)	30 (88.2)/4 (11.8)	8 (14.8)/46 (85.2)	< 0.001
Ascites (yes/no)	18 (52.9)/16 (47.1)	10 (8.5)/44 (81.5)	0.001
Gastrointestinal bleeding (yes/no)	3 (8.8)/31 (91.2)	4 (7.4)/50 (92.6)	> 0.999
Hb (g/dL)	11.6 (10.33–12.47)	12.2 (10.03–13.97)	0.225
WBC (/μL)	8900 (7200–13 600)	5750 (4800–8150)	< 0.001
PLT (×10^3^/L)	122 (84.3–144.3)	144.5 (89.5–229)	0.090
INR	1.57 (1.33–1.77)	1.08 (0.96–1.24)	< 0.001
AST (U/L)	168.5 (118–426)	155 (110.75–253)	0.237
ALT (U/L)	64 (38–153.5)	56.5 (35.25–136.25)	0.622
GGT (U/L)	424 (277–1076.75)	610.5 (380–1049.25)	0.396
T.Bil (mg/dL)	5.95 (5.05–10.28)	1.75 (1.2–2.4)	< 0.001
BUN (mg/dL)	12.5 (7.25–16)	11 (6.25–16.75)	0.699
Cre (mg/dL)	0.85 (0.62–1.2)	0.71 (0.61–0.83)	0.174
Alb (g/dL)	2.7 (2.6–3.27)	3.6 (3.2–4.1)	< 0.001
MELD	19 (17–23)	10 (8–12)	< 0.001
Benzodiazepine prophylaxis (yes/no)	21 (61.8)/13 (38.2)	29 (53.7)/25 (46.3)	0.512
Administration of prednisolone or methylprednisolone (yes/no)	8 (23.5)/26 (76.5)	0 (0.0)/54 (100.0)	< 0.001
DT (yes/no)	5 (14.7)/29 (85.3)	8 (14.8)/46 (85.2)	> 0.999

Abbreviations: ACLF, acute‐on‐chronic liver failure; Alb, albumin; ALT, alanine aminotransferase; AST, aspartate aminotransferase; BUN, blood urea nitrogen; Cre, creatinine; DT, delirium tremens; GGT, γ‐glutamyl transpeptidase; Hb, haemoglobin; INR, international normalized ratio; JAS, Japan Alcoholic Hepatitis Score; MELD, Model for End‐stage Liver Disease; PLT, platelet; T. Bil, total bilirubin; WBC, white blood cell.

The JAS, MELD and ACLF criteria have been widely used as diagnostic criteria for alcoholic hepatitis and liver failure [3, 9, 15, 17]. Furthermore, to investigate the factors affecting the survival prognosis of alcoholic hepatitis in this cohort, we applied the Cox proportional hazards model. As a result, ascites and serum creatinine levels were found to be independent prognostic factors (Table 3).

**TABLE 3:** Univariate and multivariate analyses of baseline factors associated with survival prognosis using Cox proportional hazards model.

Factor	Univariate analysis	*p* value	Multivariate analysis	*p* value
	Hazard ratio (95% CI)		Hazard ratio (95% CI)	
Sex (male/female)	4.49 (0.58–34.5)	0.149		
Age	1.04 (0.99–1.08)	0.103		
Hb (g/dL)	0.86 (0.08–4.80)	0.645		
WBC (/μL)	1.00 (1.00–1.00)	0.612		
PLT (×10^3^/μL)	0.99 (0.98–1.00)	0.014	0.99 (0.98–1.00)	0.079
PT‐INR	2.88 (1.15–7.17)	0.023	0.92 (0.24–3.51)	0.904
AST (U/L)	1.00 (1.00–1.00)	0.552		
ALT (U/L)	1.00 (0.99–1.00)	0.304		
GGT (U/L)	1.00 (1.00–1.00)	0.480		
T.Bil (mg/dL)	1.10 (1.02–1.18)	0.019	1.06 (0.95–1.19)	0.310
Cre (mg/dL)	1.90 (0.89–4.06)	0.096	3.26 (1.28–8.32)	0.013
Alb (g/dL)	0.21 (0.08–0.54)	0.001	0.65 (0.20–2.14)	0.480
Gastrointestinal bleeding (yes/no)	1.62 (0.21–12.56)	0.645		
Ascites (yes/no)	8.95 (2.32–34.54)	0.001	13.46 (2.06–87.68)	0.007

Abbreviations: Alb, albumin; ALT, alanine aminotransferase; AST, aspartate aminotransferase; CI, confidence interval; Cre, creatinine; DT, delirium tremens; GGT, γ‐glutamyl transpeptidase; Hb, haemoglobin; INR, international normalized ratio; PLT, platelet; T. Bil, total bilirubin; WBC, white blood cell.

### 3.3 No Significant Association Between DT Onset and Survival Prognosis

**FIGURE 2:**
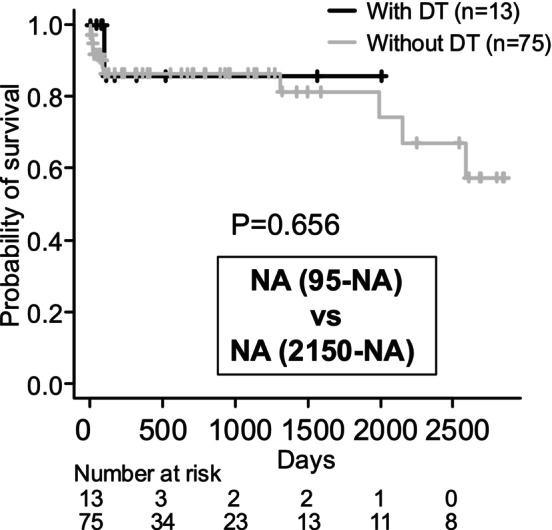
Kaplan–Meier survival curves stratified by the presence or absence of delirium tremens (DT). The MST in the group with DT was NA, with a 95% CI of 95 days to NA. In the group without DT, the MST was NA, with a 95% CI of 2150 days to NA. The log‐rank test yielded a *p* value of 0.656. Note: NA indicates that the median survival time was not reached within the observation period. DT, delirium tremens; MST, median survival time; NA, not applicable; CI, confidence interval.

DT developed in 13 patients. No significant difference was observed in the fulfilment of ACLF diagnostic criteria between patients who developed DT and those who did not (Table 4). Patients with DT had significantly lower platelet counts, and fewer received prophylactic oral benzodiazepines (Table 4). No adverse events were associated with prophylactic benzodiazepine use. Treatment of DT required sedation with midazolam or propofol in 7 of the 13 patients. Survival curves showed no significant difference in prognosis based on the presence or absence of DT (Figure 2); however, it should be noted that the observation period tended to be shorter in patients who developed DT. Hepatocellular carcinoma was observed in two patients without DT, with the first observation at 241 and 1005 days, respectively. During the follow‐up period, 10 of all patients (11.3%)—1 DT (7.7%) patient and 9 non‐DT (12.0%) patients—were confirmed to have attended a specialized alcoholism program at another hospital. Nine of those 10 were referred to the specialized program because they resumed drinking soon after discharge. One of them was a patient who was transferred to a specialized hospital because his tremor delirium was difficult to control. Only one patient was confirmed to have continued to abstain from alcohol after attending the specialized program.

**TABLE 4:** Baseline characteristics with or without DT. *n* (%), median (IQR). Fisher's exact test was performed for categorical variables and Mann–Whitney's *U* test for continuous variables.

	With DT (*n* = 13)	Without DT (*n* = 75)	*p* value
Sex (male/female)	11 (84.6)/2 (15.4)	57 (76.0)/18 (24.0)	0.724
Age	46 (37–64)	56 (45.5–65.5)	0.150
Diagnostic criteria for ACLF (ACLF/Extended/Probable/Extended Probable/without ACLF)	0 (0.0)/0 (0.0)/1 (7.7)/4 (30.8)/8 (61.5)	6 (8.0)/2 (2.7)/6 (8.0)/15 (20.0)/46 (61.3)	0.829
JAS (≥ 8/≤ 7)	8 (61.5)/5 (38.5)	30 (40.0)/45 (60.0)	0.225
GI bleeding (+/−)	1 (7.7)/12 (92.3)	6 (8.0)/69 (92.0)	> 0.999
Ascites (+/−)	4 (30.8)/9 (69.2)	24 (32.0)/51 (68.0)	> 0.999
Hb (g/dL)	13.5 (10.9–16)	11.6 (10.2–13.35)	0.138
WBC (/μL)	7200 (3800–13 500)	7200 (5200–9700)	0.737
PLT (×10^3^/L)	91 (71–138)	138 (92.5–202.5)	0.038
INR	1.08 (0.98–1.3)	1.25 (1.00–1.50)	0.236
AST (U/L)	199 (131–435)	150 (109.5–273.5)	0.153
ALT (U/L)	67 (57–162)	56 (34–137)	0.24
GGT (U/L)	1072 (424–1291)	533 (304.5–968)	0.137
T.Bil (mg/dL)	2.4 (1.8–5)	2.5 (1.4–5.45)	0.576
BUN (mg/dL)	15 (11–17)	10 (6.5–16)	0.129
Cre (mg/dL)	0.72 (0.65–1.2)	0.74 (0.61–0.99)	0.410
Alb (g/dL)	3.9 (2.7–4.6)	3.3 (2.7–3.85)	0.115
MELD	14 (9–18)	12 (9–18)	0.742
Benzodiazepine prophylaxis (yes/no)	3 (23.1)/10 (76.9)	47 (62.7)/28 (37.3)	0.013
Administration of prednisolone or methylprednisolone (yes/no)	1 (7.7)/12 (92.3)	7 (9.3)/68 (90.7)	> 0.999

Abbreviations: ACLF, acute‐on‐chronic liver failure; Alb, albumin; ALT, alanine aminotransferase; AST, aspartate aminotransferase; BUN, blood urea nitrogen; Cre, creatinine; DT, delirium tremens; GGT, γ‐glutamyl transpeptidase; Hb, haemoglobin; INR, international normalized ratio; JAS, Japan Alcoholic Hepatitis Score; MELD, Model for End‐stage Liver Disease; PLT, platelet; T. Bil, total bilirubin; WBC, white blood cell.

### 3.4 Clinical Factors Predicting the Development of DT

Table 5 presents the results of univariate and multivariate logistic regression analyses for factors associated with the development of DT. In the multivariate analysis, no prophylactic oral benzodiazepine use, low platelet count and elevated GGT levels were identified as significant predictors for the development of DT.

**TABLE 5:** Univariate and multivariate analyses of factors associated with the development of DT using logistic regression analysis.

Factor	Univariate analysis	*p* value	Multivariate analysis	*p* value
	Odds ratio		Odds ratio	
Sex (male/female)	1.74 (0.35–8.58)	0.500		
Age	0.97 (0.92–1.01)	0.140		
ACLF or related conditions (yes/no)	1.01 (0.301–3.38)	0.989		
JAS	0.429 (0.13–1.41)	0.163		
Benzodiazepine prophylaxis (yes/no)	5.60 (1.42–22.10)	0.014	8.93 (1.74–45.8)	0.009
Hb (g/dL)	1.25 (0.96–1.62)	0.096	1.23 (0.84–1.79)	0.280
WBC (/μL)	1.00 (1.00–1.00)	0.870		
PLT (×10^3^/μL)	0.99 (0.98–1.00)	0.042	0.98 (0.97–1.00)	0.017
INR	0.41 (0.06–2.58)	0.340		
AST (U/L)	1.00 (1.00–1.00)	0.130		
ALT (U/L)	1.00 (1.00–1.00)	0.660		
GGT (U/L)	1.00 (1.00–1.00)	0.055	1.00 (1.00–1.00)	0.041
T.Bil (mg/dL)	1.01 (0.89–1.14)	0.930		
BUN (mg/dL)	1.01 (0.96–1.07)	0.620		
Cre (mg/dL)	1.54 (0.64–3.74)	0.340		
Alb (g/dL)	2.09 (0.94–4.67)	0.072	1.61 (0.516–5.03)	0.412

Abbreviations: ACLF, acute‐on‐chronic liver failure; Alb, albumin; ALT, alanine aminotransferase; AST, aspartate aminotransferase; BUN, blood urea nitrogen; Cre, creatinine; DT, delirium tremens; GGT, γ‐glutamyl transpeptidase; Hb, haemoglobin; INR, international normalized ratio; JAS, Japan Alcoholic Hepatitis Score; PLT, platelet; T. Bil, total bilirubin; WBC, white blood cell.

### 3.5 Impact of Delirium Tremens (DT) on Subsequent Follow‐Up

**FIGURE 3:**
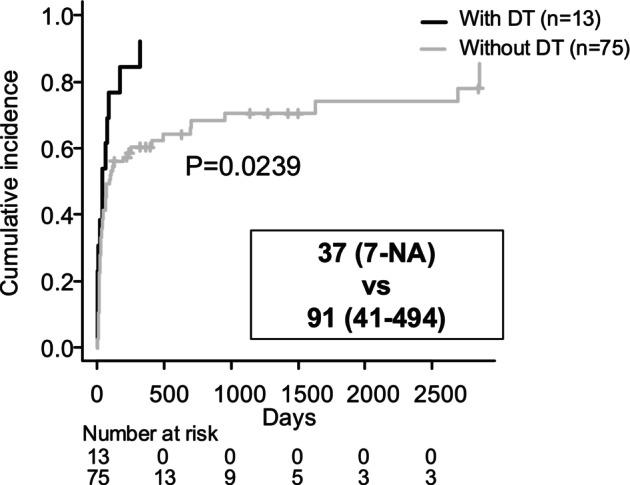
Analysis of follow‐up time using a competing risk model, with death as a competing event, stratified by the presence or absence of delirium tremens (DT). In the group with DT, the median follow‐up time was 37 days (95% CI 7 days to NA). In the group without DT, the median follow‐up time was 91 days (95% CI 41 days to 494 days). The comparison was evaluated using Grey's test, with a *p* value of 0.0239. Note: NA indicates that the upper limit of the confidence interval could not be determined within the observation period. DT, delirium tremens; CI, confidence interval; NA, not applicable.

Analysis of the time to the end of follow‐up using a competing risk model, with death as a competing risk, showed that follow‐up was significantly shorter in patients who developed DT (Figure 3, 37 days vs. 91 days, *p* = 0.0239). The status of follow‐up after hospital discharge is shown in Table 6. Significantly fewer patients with DT continued follow‐up at their original (our) hospital. Overall, 50 patients continued follow‐up at the original hospital post‐discharge, and 36 of them (72%) remained sober during the follow‐up period (Table 7). Of the 65 individuals whose follow‐up ended at the original hospital after discharge or after outpatient care, 22 (33.6%) returned for unscheduled visits due to alcohol‐related health issues (Table 8).

**TABLE 6:** The status of follow‐up after discharge from hospital. *n* (%). Fisher's exact test was performed. Using Fisher's exact test, a significant difference was observed overall between the presence and absence of DT (*p* = 0.003). In the post hoc analysis with Bonferroni correction, a significant difference was noted between ‘Same hospital’ and ‘Hospital transfer’ (*p* = 0.013).

		Status of initial follow‐up after discharge					
Group	Same hospital	Unauthorized interruption	Referral to other hospitals	Hospital transfer	Death in hospital	Total	*p* value
With DT	3 (23.1)	2 (15.4)	3 (23.1)	4 (30.8)	1 (7.7)	13 (100)	0.003
Without DT	47 (62.7)	5 (6.7)	14 (18.7)	2 (2.7)	7 (9.3)	75 (100)	

**TABLE 7:** Sobriety during follow‐up (*n*). Fisher's exact test was performed.

	Sobriety during follow‐up		
Group	Yes	No	*p* value
With DT	3	0	0.550
Without DT	33	14	

Abbreviation: DT, delirium tremens.

**TABLE 8:** Harmful drinking after the end of follow‐up (*n*). Fisher's exact test was performed.

	Harmful drinking after the end of follow‐up		
Group	Yes	No	*p* value
With DT	**3**	**9**	**0.737**
Without DT	**19**	**34**	

Abbreviation: DT, delirium tremens.

**FIGURE 4:**
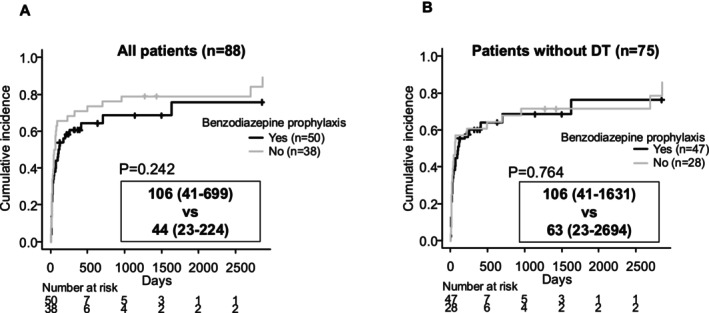
Analysis of follow‐up time using a competing risk model, with death as a competing event, stratified by the use or non‐use of benzodiazepine prophylaxis. Analysis of all patients. In the group receiving benzodiazepine prophylaxis, the median follow‐up time was 106 days (95% CI 41 days to 699 days). In the group without prophylaxis, the median follow‐up time was 44 days (95% CI 23 days to 224 days). The comparison was evaluated using Grey's test, with a *p* value of 0.242. Analysis of patients who did not develop delirium tremens (DT). In the group receiving benzodiazepine prophylaxis, the median follow‐up time was 106 days (95% CI 41 days to 1631 days). In the group without prophylaxis, the median follow‐up time was 63 days (95% CI 23 days to 2694 days). The comparison was evaluated using Grey's test, with a *p* value of 0.764. DT, delirium tremens; CI, confidence interval.

Finally, we examined the effect of benzodiazepine prophylaxis on the duration of follow‐up. Although there was a slight tendency for follow‐up duration to be longer in patients who received benzodiazepine prophylaxis, this difference was not statistically significant (Figure 4A). Furthermore, when considering only patients who did not develop DT, the presence or absence of benzodiazepine prophylaxis had no noticeable effect on follow‐up duration (Figure 4B). The combined findings from Figures 3 and 4 indicate that benzodiazepine prophylaxis in high‐risk patients for DT not only helps to prevent DT onset (Table 4) but also eliminates the DT‐associated shortening of the follow‐up period. This suggests that DT itself, rather than differences in underlying DT risk factors (which were addressed by prophylactic treatment), was the critical factor in shortening the follow‐up period.

## 4 Discussion

### 4.1 ACLF Stratifies the Survival Prognosis of Alcoholic Hepatitis

The present study showed that survival prognosis in alcoholic hepatitis patients can be stratified by whether they meet the Japanese diagnostic criteria for ACLF or related conditions. This finding was expected, as the somewhat different criteria for ACLF defined by the European Association for the Study of the Liver‐Chronic Liver Failure Consortium have also been identified as a poor prognostic factor for alcoholic hepatitis [18, 19]. On the other hand, the overall Japanese Alcoholic Score (JAS)—used to assess the severity of alcoholic hepatitis in Japan—did not significantly stratify prognosis in our study. Notably though, the white blood cell count, a component of JAS, was significantly higher in patients who met the criteria for ACLF or related conditions. Leukaemic infiltration is associated with the severity of alcoholic hepatitis [20], and granulocyte apheresis therapy has been used, mainly in Japan, with good results reported [21]. As white blood cell counts are included in the JAS, the JAS may still be useful in determining treatment options.

In addition, application of the Cox proportional hazards model to the current cohort identified ascites and serum creatinine levels as independent prognostic factors. Serum creatinine is also a component of the JAS and MELD scores. Additionally, in the ACLF criteria, creatinine levels are used to help diagnose organ failure and classify severity [2]. However, ascites is not included in these criteria. Incorporating ascites as a prognostic factor in future models may potentially improve outcome prediction systems for alcoholic hepatitis.

### 4.2 Management of Delirium Tremens (DT)

We observed that the onset of DT was not statistically associated with ACLF or related conditions diagnosis, or with survival, but fewer patients with DT continued follow‐up in the same hospital post‐discharge. Although the study could not identify the causes, it is plausible that doctors unfamiliar with the management of alcohol withdrawal, including DT, may have had difficulty in establishing sufficient rapport with the patients to facilitate follow‐up.

In contrast, another study reported that DT is associated with a high mortality rate [22]. One possible reason for this difference is that our cohort consisted solely of patients with alcohol‐related liver disease. Additionally, in our study, the onset of DT shortened the follow‐up period, and we may have missed the death of high‐risk patients who should have been followed up continuously. This highlights the importance of not only preventing DT onset in alcohol‐dependent patients but also ensuring continuous follow‐up with patients through initiatives such as inter‐professional collaboration.

In our study, 15% of alcoholic hepatitis patients developed DT during hospitalization, which may be comparable to a study in the United States in which 18% of alcoholic hepatitis patients required treatment for withdrawal symptoms [23].

The annual incidence of hepatocellular carcinoma in alcohol‐related cirrhosis has been reported to be approximately 2% [24]. The finding of two cases of carcinogenesis in this study cohort, which included patients who had not necessarily developed cirrhosis, is noteworthy. The short follow‐up of patients who developed DT may have missed important events in these patients, including hepatocellular carcinoma. This underscores the argument that effective management of alcohol withdrawal syndrome is essential in alcoholic hepatitis care, ensuring follow‐up continuity.

We identified elevated GGT levels and low platelet counts as risk factors for DT in alcoholic hepatitis patients. Elevated GGT levels may reflect previous alcohol consumption [17] and have been associated with the development of alcohol withdrawal syndrome [25]. Low platelet counts were already known as a risk factor for DT [26].

Importantly, we found that oral benzodiazepine therapy is effective and safe in reducing the risk of DT, even in alcoholic hepatitis cases managed by hepatologists. The Prediction of Alcohol Withdrawal Severity Scale (PAWSS) [27] and the Clinical Institute Withdrawal Assessment scale for Alcohol, revised (CIWA‐Ar), are valuable tools for deciding on the prophylactic use of benzodiazepine [12, 28]. Additionally, the CIWA‐Ar helps to reduce unnecessary benzodiazepine dosage [29, 30]. However, due to challenges in obtaining accurate histories in the acute phase, differentiating withdrawal symptoms from hepatic encephalopathy, and the complexity of scoring, these assessment tests were not performed in the present study, presumably reflecting a common situation in Japanese healthcare organizations. It is noteworthy that, even under these circumstances, benzodiazepine administration was shown to be useful and safe in the prevention of DT in our study. However, although no adverse events were recorded in this study, the possibility of benzodiazepine‐induced drowsiness or dizziness cannot be ruled out. Multidisciplinary collaboration, particularly including nurses, may be important, as it is difficult for an hepatologist alone to perform an assessment using PAWSS or CIWA‐Ar [31, 32].

### 4.3 Patient Relapse During and After Follow‐Up

Reports on the long‐term survival prognosis of alcoholic hepatitis are limited, but have indicated that sustained sobriety post‐improvement is a crucial factor [33]. Hence, managing alcoholic hepatitis necessitates not only assessing disease severity at onset and during treatment but also ensuring continuous follow‐up for sobriety.

Notably, only 28% of patients relapsed during follow‐up at their original (our) hospital, regardless of DT presence in this study. However, after discontinuation of follow‐up, 36% of the patients were confirmed to have experienced recurrent alcohol‐related health problems. This percentage excludes patients with recurring drinking‐related problems who did not visit our hospital for those problems, either because they did not seek care or visited other healthcare providers. A previous study also showed that follow‐up in the same facility where the patient was admitted—regardless of whether or not the patient participated in a specialized alcoholism program—helps to prevent relapse [34]. In particular, our data suggest that follow‐up by hepatologists at the hospital in charge of inpatient care can help prevent relapse. Patients with alcoholic hepatitis have a favourable prognosis if they maintain abstinence. While combining the management of liver disease with specialized alcoholism programs is ideal [8, 31], a similar level of care is difficult to achieve in medical centers without such programs. In this study, only about 10% of patients participated in specialized alcoholism programs at another hospital during the observation period. Many patients refuse participation, and pressuring them may deteriorate the doctor–patient relationship, leading to worse prognoses due to loss of follow‐up.

### 4.4 Care Organization: Role of Multidisciplinary Care and Hepatologist Education

Hepatologists need to be able to manage alcohol‐related liver disease and alcohol use disorders, including severe alcohol withdrawal syndrome [35]. Particularly in Japan, where single‐specialty psychiatric units are predominant [4], there is a treatment gap because hospitals that manage alcohol‐related liver disease and those that manage alcohol use disorders are separate. Integrated and collaborative models that streamline care for both alcohol‐related liver disease and alcohol use disorders are a promising approach to closing this treatment gap [36]. Hepatologists are expected to actively address this issue in collaboration with other professionals, such as addiction medicine providers and social workers [35].

Recent attention has focused on screening and brief interventions by primary care physicians for patients with alcohol use disorders [37]. Several reports have demonstrated the effectiveness of brief interventions in reducing alcohol consumption [38, 39]. In Japan, the alcohol reduction drug nalmefene has recently been prescribed even by physicians who are not specialists in alcohol abuse disorders [40]. It is expected that alcohol abuse management by hepatologists, including brief intervention, will be widely implemented and that patients will benefit from continued follow‐up, including alcohol reduction treatment. Although improvement in the prognosis of alcohol‐related liver injury with reduced (and not fully abstained) alcohol consumption is currently unproven, it is hoped that patients will benefit from continued therapeutic intervention by hepatologists in combination with brief intervention [41, 42].

Although the follow‐up period was limited, the 28% relapse rate during follow‐up at our hospital appears to compare favourably with other studies in several countries reporting > 60% relapse [18, 23, 33, 43]. Given the potential of psychosocial interventions to improve cases of alcoholism‐induced liver disease is well established [44, 45], we expect that the introduction of psychosocial interventions by hepatologists may help prevent patients from dropping out and further improve patient behaviour.

### 4.5 Limitations of our Study

The main limitation of this study is that it is a single‐centre, retrospective study, which likely introduces various biases, particularly given the lack of liver tissue diagnosis. Most cases meeting the diagnostic criteria for ACLF or related conditions had unknown baseline Child–Pugh scores, and potentially represented a mix of different conditions. This reflects the reality of alcoholic hepatitis care, where alcohol‐related liver disease is not always well monitored. Furthermore, assessments and treatments of alcoholism may be different in general hospitals with psychiatrists and/or other specialists, but a direct comparison with such situations was not made. Finally, the study's reliance on medical records made it impossible to determine the exact amount of alcohol consumed, the duration of drinking, and the history of treatment for alcohol dependence, all representing critical information for assessing the risk of resuming harmful drinking [33, 46]. However, obtaining this information from patients remains a challenge in practice. Expanding brief interventions on alcohol use in primary care and hospital wards may facilitate obtaining this information in daily practice and provide greater insight into the pathogenesis and prognosis of alcohol‐related liver disease.

## 5 Conclusions

Our retrospective study of alcoholic hepatitis patients in our psychiatry‐absent hospital found the expected decrease in survival prognosis associated with the diagnosis ‘ACLF or related conditions’. Although hepatologists appear to manage the various aspects of treatment well, as long as the patients remain under their guidance, there was a tendency to break off contact, especially in patients who developed DT. Given the frequent relapses after follow‐up discontinuation, this suggests that a more active involvement of hepatologists in planning long‐term management, including alcohol use disorder medications and referral to recovery supports, could potentially improve outcomes.

## Ethics Statement

All study protocols were reviewed and approved by the ethics committee at Fujita Health University School of Medicine (approval no. HM23‐213) and were conducted in accordance with the 1975 Declaration of Helsinki. Written informed consent treatment had been obtained from each patient, but the need for informed consent for participation in this study was waived because of the retrospective design.

## Consent

Written informed consent for treatment had been obtained from each patient, but the need for informed consent for participation in this study was waived because of the retrospective design.

## Conflicts of Interest

The authors declare no conflicts of interest.

## Permission to Reproduce Material From Other Sources

Not applicable.

## Data Availability

The data that support the findings of this study are available from the corresponding author upon reasonable request.

## References

[1] H. Enomoto , N. Akuta , H. Hikita , et al., “Etiological Changes of Liver Cirrhosis and Hepatocellular Carcinoma‐Complicated Liver Cirrhosis in Japan: Updated Nationwide Survey From 2018 to 2021,” Hepatology Research 54, no. 8 (2024): 763–772. 38638067 10.1111/hepr.14047

[2] S. Mochida , N. Nakayama , S. Terai , et al., “Diagnostic Criteria for Acute‐On‐Chronic Liver Failure and Related Disease Conditions in Japan,” Hepatology Research 52, no. 5 (2022): 417–421. 35591813 10.1111/hepr.13763

[3] N. Nakayama , H. Uemura , Y. Uchida , et al., “A Multicenter Pilot Survey to Clarify the Clinical Features of Patients With Acute‐On‐Chronic Liver Failure in Japan,” Hepatology Research 48, no. 4 (2018): 303–312. 29341357 10.1111/hepr.13064

[4] Japanese Ministry of Health, Labour and Welfare 2022. A Survey of Medical Facilities.

[5] Y. Osaki , A. Kinjo , S. Higuchi , et al., “Prevalence and Trends in Alcohol Dependence and Alcohol Use Disorders in Japanese Adults; Results From Periodical Nationwide Surveys,” Alcohol and Alcoholism 51, no. 4 (2016): 465–473. 26873982 10.1093/alcalc/agw002

[6] G, Ayares , F, Idalsoaga , LA, Díaz , J, Arnold , JP, Arab . Current Medical Treatment for Alcohol‐Associated Liver Disease. Journal of Clinical and Experimental Hepatology 2022;12(5):1333. Available from: /pmc/articles/PMC9499849/. 36157148 10.1016/j.jceh.2022.02.001 PMC9499849

[7] D. W. Crabb , R. Bataller , N. P. Chalasani , et al., “Standard Definitions and Common Data Elements for Clinical Trials in Patients With Alcoholic Hepatitis: Recommendation From the NIAAA Alcoholic Hepatitis Consortia,” Gastroenterology 150, no. 4 (2016): 785–790. 26921783 10.1053/j.gastro.2016.02.042 PMC5287362

[8] M. A. Schuckit , “Alcohol‐Use Disorders,” Lancet 373, no. 9662 (2009): 492–501. 19168210 10.1016/S0140-6736(09)60009-X

[9] A. K. Singal , R. Bataller , J. Ahn , P. S. Kamath , and V. H. Shah , “ACG Clinical Guideline: Alcoholic Liver Disease,” American Journal of Gastroenterology 113, no. 2 (2018): 175–194. 29336434 10.1038/ajg.2017.469 PMC6524956

[10] C. D. Lansford , C. H. Guerriero , M. J. Kocan , et al., “Improved Outcomes in Patients With Head and Neck Cancer Using a Standardized Care Protocol for Postoperative Alcohol Withdrawal,” Archives of Otolaryngology – Head & Neck Surgery 134, no. 8 (2008): 865–872. 18711062 10.1001/archotol.134.8.865

[11] J. P. Reoux , C. A. Malte , D. R. Kivlahan , and A. J. Saxon , “The Alcohol Use Disorders Identification Test (AUDIT) Predicts Alcohol Withdrawal Symptoms During Inpatient Detoxification,” Journal of Addictive Diseases 21, no. 4 (2002): 81–91. 12296504 10.1300/J069v21n04_08

[12] P. Naik and J. Lawton , “Pharmacological Management of Alcohol Withdrawal: A Meta‐Analysis and Evidence‐Based Practice Guideline,” Journal of the American Medical Association 278, no. 2 (1997): 144–151. 9214531 10.1001/jama.278.2.144

[13] M. F. Mayo‐Smith , L. H. Beecher , T. L. Fischer , et al., “Management of Alcohol Withdrawal Delirium: An Evidence‐Based Practice Guideline,” Archives of Internal Medicine 164, no. 13 (2004): 1405–1412. 15249349 10.1001/archinte.164.13.1405

[14] The ASAM Clinical Practice Guideline on Alcohol Withdrawal Management,” Journal of Addiction Medicine 14, no. 3S (2020): 1–72. 10.1097/ADM.0000000000000668 32511109

[15] Y. Horie , H. Ebinuma , M. Kikuchi , and T. Kanai , “Current Status of Alcoholic Liver Disease in Japan and Therapeutic Strategy,” Nihon Arukōru Yakubutsu Igakkai Zasshi 51, no. 2 (2016): 71–90. 30462383

[16] Y. Kanda , “Investigation of the Freely Available Easy‐To‐Use Software “EZR” for Medical Statistics,” Bone Marrow Transplantation 48, no. 3 (2013): 452–458. 23208313 10.1038/bmt.2012.244 PMC3590441

[17] M. Thursz , A. Gual , C. Lackner , et al., “EASL Clinical Practice Guidelines: Management of Alcohol‐Related Liver Disease,” Journal of Hepatology 69, no. 1 (2018): 154–181. 29628280 10.1016/j.jhep.2018.03.018

[18] J. Gratacós‐Ginès , P. Ruz‐Zafra , M. Celada‐Sendino , et al., “Recurrent Alcohol‐Associated Hepatitis Is Common and Is Associated With Increased Mortality,” Hepatology 80, no. 3 (2024): 621–632. 38441908 10.1097/HEP.0000000000000825

[19] S. M. Rutledge , R. Nathani , B. E. Wyatt , et al., “Age Added to MELD or ACLF Predicts Survival in Patients With Alcohol‐Associated Hepatitis Declined for Liver Transplantation,” Hepatology Communications 8, no. 9 (2024): e0514. 39167426 10.1097/HC9.0000000000000514 PMC11340926

[20] J. Ma , A. Guillot , Z. Yang , et al., “Distinct Histopathological Phenotypes of Severe Alcoholic Hepatitis Suggest Different Mechanisms Driving Liver Injury and Failure,” Journal of Clinical Investigation 132, no. 14 (2022): e157780. 35838051 10.1172/JCI157780 PMC9282929

[21] R. Kasuga , P. s. Chu , N. Taniki , et al., “Granulocyte‐Monocyte/Macrophage Apheresis for Steroid‐Nonresponsive or Steroid‐Intolerant Severe Alcohol‐Associated Hepatitis: A Pilot Study,” Hepatology Communications 8, no. 2 (2024): e0371. 38285891 10.1097/HC9.0000000000000371 PMC10830070

[22] J. G. Bramness , I. H. Heiberg , A. Høye , and I. Rossow , “Mortality and Alcohol‐Related Morbidity in Patients With Delirium Tremens, Alcohol Withdrawal State or Alcohol Dependence in Norway: A Register‐Based Prospective Cohort Study,” Addiction 118, no. 12 (2023): 2352–2359. 37465900 10.1111/add.16297

[23] K. R. Patidar , M. G. Ortiz , J. E. Slaven , et al., “Incidence, Clinical Characteristics, and Risk Factors Associated with Recurrent Alcohol‐Associated Hepatitis,” Hepatology Communications 7, no. 12 (2023): e0341, 10.1097/HC9.0000000000000341. 38055648 PMC10984669

[24] K. Tarao , A. Nozaki , T. Ikeda , et al., “Real Impact of Liver Cirrhosis on the Development of Hepatocellular Carcinoma in Various Liver Diseases—Meta‐Analytic Assessment,” Cancer Medicine 8, no. 3 (2019): 1054–1065. 30791221 10.1002/cam4.1998 PMC6434205

[25] E. Wood , L. Albarqouni , S. Tkachuk , et al., “Will This Hospitalized Patient Develop Severe Alcohol Withdrawal Syndrome?: The Rational Clinical Examination Systematic Review,” Journal of the American Medical Association 320, no. 8 (2018): 825–833. 30167704 10.1001/jama.2018.10574 PMC6905615

[26] C. M. Goodson , B. J. Clark , and I. S. Douglas , “Predictors of Severe Alcohol Withdrawal Syndrome: A Systematic Review and Meta‐Analysis,” Alcoholism, Clinical and Experimental Research 38, no. 10 (2014): 2664–2677. 25346507 10.1111/acer.12529

[27] J. R. Maldonado , Y. Sher , J. F. Ashouri , et al., “The “Prediction of Alcohol Withdrawal Severity Scale” (PAWSS): Systematic Literature Review and Pilot Study of a new Scale for the Prediction of Complicated Alcohol Withdrawal Syndrome,” Alcohol 48, no. 4 (2014): 375–390. 24657098 10.1016/j.alcohol.2014.01.004

[28] J. T. Sullivan , K. Sykora , J. Schneiderman , C. A. Naranjo , and E. M. Sellers , “Assessment of Alcohol Withdrawal: The Revised Clinical Institute Withdrawal Assessment for Alcohol Scale (CIWA‐Ar),” British Journal of Addiction 84, no. 11 (1989): 1353–1357. 2597811 10.1111/j.1360-0443.1989.tb00737.x

[29] M. F. Mayo‐Smith , “Pharmacological Management of Alcohol Withdrawal. A Meta‐analysis and Evidence‐Based Practice Guideline American Society of Addiction Medicine Working Group on Pharmacological Management of Alcohol Withdrawal,” JAMA 278, no. 2 (1997): 144–151, 10.1001/jama.1997.03550020076042. 9214531

[30] R. Saitz , M. F. Mayo‐Smith , M. S. Roberts , H. A. Redmond , D. R. Bernard , and D. R. Calkins , “Individualized Treatment for Alcohol Withdrawal. A Randomized Double‐Blind Controlled Trial,” JAMA 272, no. 7 (1994): 519–523. 8046805

[31] G. Addolorato , A. Mirijello , P. Barrio , and A. Gual , “Treatment of Alcohol use Disorders in Patients With Alcoholic Liver Disease,” Journal of Hepatology 65, no. 3 (2016): 618–630. 27155530 10.1016/j.jhep.2016.04.029

[32] K. J. Moriarty , “Review: Alcohol Care Teams: Where Are We Now?,” Frontline Gastroenterology 11, no. 4 (2020): 293. 32582422 10.1136/flgastro-2019-101241 PMC7307041

[33] J. Altamirano , H. López‐Pelayo , J. Michelena , et al., “Alcohol Abstinence in Patients Surviving an Episode of Alcoholic Hepatitis: Prediction and Impact on Long‐Term Survival,” Hepatology 66, no. 6 (2017): 1842–1853. 28646515 10.1002/hep.29338

[34] H. López‐Pelayo , L. Miquel , J. Altamirano , et al., “Treatment Retention in a Specialized Alcohol Programme After an Episode of Alcoholic Hepatitis: Impact on Alcohol Relapse,” Journal of Psychosomatic Research 116 (2019): 75–82. 30654998 10.1016/j.jpsychores.2018.11.020

[35] J. A. Ratner , H. Blaney , and D. A. Rastegar , “Management of Alcohol Withdrawal Syndrome in Patients With Alcohol‐Associated Liver Disease,” Hepatology Communications 8, no. 2 (2024): e0372. 38251886 10.1097/HC9.0000000000000372 PMC10805424

[36] L. Y. Haque and L. Leggio , “Integrated and Collaborative Care Across the Spectrum of Alcohol‐Associated Liver Disease and Alcohol Use Disorder,” Hepatology 80, no. 6 (2024): 1408–1423. 38935926 10.1097/HEP.0000000000000996 PMC11841743

[37] S. J. Curry , A. H. Krist , D. K. Owens , et al., “Screening and Behavioral Counseling Interventions to Reduce Unhealthy Alcohol Use in Adolescents and Adults: US Preventive Services Task Force Recommendation Statement,” JAMA 320, no. 18 (2018): 1899–1909, 10.1001/jama.2018.16789. 30422199

[38] E. F. S. Kaner , H. O. Dickinson , F. Beyer , et al., “The Effectiveness of Brief Alcohol Interventions in Primary Care Settings: A Systematic Review,” Drug and Alcohol Review 28, no. 3 (2009): 301–323. 19489992 10.1111/j.1465-3362.2009.00071.x

[39] M. F. Fleming , M. P. Mundt , M. T. French , L. B. Manwell , E. A. Stauffacher , and K. L. Barry , “Brief Physician Advice for Problem Drinkers: Long‐Term Efficacy and Benefit‐Cost Analysis,” Alcoholism, Clinical and Experimental Research 26, no. 1 (2002): 36–43. 11821652

[40] N. Hara , A. Hiraoka , M. Nakai , et al., “Brief Intervention for Chronic Liver Disease Patients With Alcohol Use Disorder in a Hepatology Outpatient Unit: Effects and Limitations,” Hepatology Research 54, no. 11 (2024): 1099–1105. 38801372 10.1111/hepr.14060

[41] K. Charlet and A. Heinz , “Harm Reduction—A Systematic Review on Effects of Alcohol Reduction on Physical and Mental Symptoms,” Addiction Biology 22, no. 5 (2017): 1119–1159. 27353220 10.1111/adb.12414

[42] H. Yoshiji , S. Nagoshi , T. Akahane , et al., “Evidence‐Based Clinical Practice Guidelines for Liver Cirrhosis 2020,” Hepatology Research 51, no. 7 (2021): 725–749. 34228859 10.1111/hepr.13678

[43] J. R. Potts , S. Goubet , M. A. Heneghan , and S. Verma , “Determinants of Long‐Term Outcome in Severe Alcoholic Hepatitis,” Alimentary Pharmacology & Therapeutics 38, no. 6 (2013): 584–595. 23879720 10.1111/apt.12427

[44] S. Rogal , A. Youk , H. Zhang , et al., “Impact of Alcohol Use Disorder Treatment on Clinical Outcomes Among Patients With Cirrhosis,” Hepatology 71, no. 6 (2020): 2080–2092. 31758811 10.1002/hep.31042 PMC8032461

[45] M. A. Elfeki , M. A. Abdallah , L. Leggio , and A. K. Singal , “Simultaneous Management of Alcohol Use Disorder and Liver Disease: A Systematic Review and Meta‐Analysis,” Journal of Addiction Medicine 17, no. 2 (2023): E119–E128. 36259647 10.1097/ADM.0000000000001084 PMC12458788

[46] A. De Gottardi , L. Spahr , P. Gelez , et al., “A Simple Score for Predicting Alcohol Relapse After Liver Transplantation: Results From 387 Patients Over 15 Years,” Archives of Internal Medicine 167, no. 11 (2007): 1183–1188. 17563028 10.1001/archinte.167.11.1183

